# Supplementary material for: FastqPuri: high-performance preprocessing of RNA-seq data
Source: BMC Bioinformatics. 2019 May 3;20:226. doi: 10.1186/s12859-019-2799-0 (PMC6500068; doi:10.1186/s12859-019-2799-0)
Supplement: Supplementary file 2 — Archive of FastqPuri. Archive containing all files needed to install and run FastqPuri v1.0.6. Date stamp March 22, 2019. (GZ 47,819 kb) [file 12859_2019_2799_MOESM2_ESM.gz › FastqPuri-1.0.6/html/trim_8h_source.html]

FastqPuri: include/trim.h Source File


FastqPuri


- include

trim.h

Go to the documentation of this file.

1 /\*\*\*\*\*\*\*\*\*\*\*\*\*\*\*\*\*\*\*\*\*\*\*\*\*\*\*\*\*\*\*\*\*\*\*\*\*\*\*\*\*\*\*\*\*\*\*\*\*\*\*\*\*\*\*\*\*\*\*\*\*\*\*\*\*\*\*\*\*\*\*\*\*\*\*\*

2  \* Copyright (C) 2017 by Paula Perez Rubio \*

3  \* \*

4  \* This file is part of FastqPuri. \*

5  \* \*

6  \* FastqPuri is free software: you can redistribute it and/or modify \*

7  \* it under the terms of the GNU General Public License as \*

8  \* published by the Free Software Foundation, either version 3 of the \*

9  \* License, or (at your option) any later version. \*

10  \* \*

11  \* FastqPuri is distributed in the hope that it will be useful, \*

12  \* but WITHOUT ANY WARRANTY; without even the implied warranty of \*

13  \* MERCHANTABILITY or FITNESS FOR A PARTICULAR PURPOSE. See the \*

14  \* GNU General Public License for more details. \*

15  \* \*

16  \* You should have received a copy of the GNU General Public License \*

17  \* along with FastqPuri. \*

18  \* If not, see <http://www.gnu.org/licenses/>. \*

19  \*\*\*\*\*\*\*\*\*\*\*\*\*\*\*\*\*\*\*\*\*\*\*\*\*\*\*\*\*\*\*\*\*\*\*\*\*\*\*\*\*\*\*\*\*\*\*\*\*\*\*\*\*\*\*\*\*\*\*\*\*\*\*\*\*\*\*\*\*\*\*\*\*\*\*\*/

20

29 #ifndef TRIM\_H\_

30 #define TRIM\_H\_

31

32 #include <stdio.h>

33 #include "Lmer.h"

34 #include "fq\_read.h"

35 #include "defines.h"

36 #include "tree.h"

37 #include "bloom.h"

38 #include "adapters.h"

39

40 int trim\_adapter(Fq\_read \*seq, Ad\_seq \*adap\_list);

41 int trim\_sequenceN(Fq\_read \*seq);

42 int trim\_sequenceQ(Fq\_read \*seq);

43 bool is\_read\_inTree(Tree \*tree\_ptr, Fq\_read \*seq);

44 bool is\_read\_inBloom(Bfilter \*tree\_ptr, Fq\_read \*seq, Bfkmer \*ptr\_Bfkmer);

45 int Qtrim\_global(Fq\_read \*seq, int left, int right, char type);

46

47 /\* static functions

48 \* static int no\_N(Fq\_read \*seq);

49 \* static int Nfree\_Lmer(Fq\_read \*seq, int minL);

50 \* static int Ntrim\_ends(Fq\_read \*seq, int minL);

51 \* static int no\_lowQ(Fq\_read \*seq, int minQ);

52 \* static int Qtrim\_ends(Fq\_read \*seq, int minQ, int minL);

53 \* static int Qtrim\_frac(Fq\_read \*seq, int minQ ,int nlowQ );

54 \* static int Qtrim\_endsfrac(Fq\_read \*seq, int minQ, int minL, int nlowQ );

55 \*/

56

57 #endif // TRIM\_H\_

trim\_sequenceQ

int trim\_sequenceQ(Fq\_read \*seq)

trims sequence based on lowQ base callings

**Definition:** trim.c:692

adapters.h

sequence manipulation for alignment

is\_read\_inBloom

bool is\_read\_inBloom(Bfilter \*tree\_ptr, Fq\_read \*seq, Bfkmer \*ptr\_Bfkmer)

checks if a read is in Bloom filter. It computes the score for the read and returns true if it exceed...

**Definition:** trim.c:734

bloom.h

functions that implement the bloom filter

\_fq\_read

stores a fastq entry

**Definition:** fq\_read.h:37

tree.h

Construction of tree, check paths, write tree, read in tree.

trim\_adapter

int trim\_adapter(Fq\_read \*seq, Ad\_seq \*adap\_list)

trims sequence based on presence of N nucleotides

**Definition:** trim.c:628

Lmer.h

Manipulation of Lmers and sequences.

defines.h

Macro definitions.

is\_read\_inTree

bool is\_read\_inTree(Tree \*tree\_ptr, Fq\_read \*seq)

check if Lread is contained in tree. It computes the score for the read and its reverse complement; i...

**Definition:** trim.c:712

\_bfilter

Bloom filter structure.

**Definition:** bloom.h:41

\_ad\_seq

stores an adapter entry

**Definition:** adapters.h:38

fq\_read.h

fastq entries manipulations (read/write)

trim\_sequenceN

int trim\_sequenceN(Fq\_read \*seq)

trims sequence based on presence of N nucleotides

**Definition:** trim.c:665

\_bfkmer

stores a processed kmer (2 bits pro nucleotide)

**Definition:** bloom.h:57

\_tree

structure containing a T\_ACGT-tree.

**Definition:** tree.h:60

Qtrim\_global

int Qtrim\_global(Fq\_read \*seq, int left, int right, char type)

trims left from the left and right from the right

**Definition:** trim.c:353


---

Generated on Mon Mar 19 2018 23:42:01 for FastqPuri by  

 1.8.14
